# Higher neuron densities in the cerebral cortex and larger cerebellums may limit dive times of delphinids compared to deep-diving toothed whales

**DOI:** 10.1371/journal.pone.0226206

**Published:** 2019-12-16

**Authors:** Sam H. Ridgway, Robert H. Brownson, Kaitlin R. Van Alstyne, Robert A. Hauser

**Affiliations:** 1 National Marine Mammal Foundation, San Diego, California, United States of America; 2 Department of Pathology, School of Medicine, University of California, San Diego, La Jolla, California, United States of America; 3 Department of Cell Biology and Human Anatomy, School of Medicine, University of California, Davis, Davis, California, United States of America; 4 National Marine Mammal Foundation, San Diego, California, United States of America; 5 Department of Neurology, University of South Florida, Tampa, Florida, United States of America; Institute of Deep-sea Science and Engineering, Chinese Academy of Sciences, CHINA

## Abstract

Since the work of Tower in the 1950s, we have come to expect lower neuron density in the cerebral cortex of larger brains. We studied dolphin brains varying from 783 to 6215g. As expected, average neuron density in four areas of cortex decreased from the smallest to the largest brain. Despite having a lower neuron density than smaller dolphins, the killer whale has more gray matter and more cortical neurons than any mammal, including humans. To begin a study of non-dolphin toothed whales, we measured a 596g brain of a pygmy sperm whale and a 2004g brain of a Cuvier’s beaked whale. We compared neuron density of Nissl stained cortex of these two brains with those of the dolphins. Non-dolphin brains had lower neuron densities compared to all of the dolphins, even the 6215g brain. The beaked whale and pygmy sperm whale we studied dive deeper and for much longer periods than the dolphins. For example, the beaked whale may dive for more than an hour, and the pygmy sperm whale more than a half hour. In contrast, the dolphins we studied limit dives to five or 10 minutes. Brain metabolism may be one feature limiting dolphin dives. The brain consumes an oversized share of oxygen available to the body. The most oxygen is used by the cortex and cerebellar gray matter. The dolphins have larger brains, larger cerebellums, and greater numbers of cortex neurons than would be expected given their body size. Smaller brains, smaller cerebellums and fewer cortical neurons potentially allow the beaked whale and pygmy sperm whale to dive longer and deeper than the dolphins. Although more gray matter, more neurons, and a larger cerebellum may limit dolphins to shorter, shallower dives, these features must give them some advantage. For example, they may be able to catch more elusive individual high-calorie prey in the upper ocean.

## Introduction

Is there an ecological advantage to having a smaller brain with less cortex and fewer neurons? This question has been asked relative to diving mammals that must search for food at depth using limited oxygen stores [1, 2]. Brains are metabolically expensive [3–5] and should not grow larger unless increased size provides some advantage. The total energetic requirement of the brain increases with increasing numbers of neurons, which leads to the need for more food and more time spent feeding to support the brain [5, 6]. Previous studies of bottlenose dolphins (*Tursiops truncatus*) demonstrated that the highest metabolism is in the gray matter of the cerebral cortex and cerebellum [7, 8]. Altogether, these findings suggest that a diving animal with a brain comprising a very small percentage of its body weight and containing fewer neurons should be able to make oxygen-limited dives for a longer period of time.

In earlier studies, only body size was positively correlated with dive time [9, 10]. However, recent work has shown that some toothed whales (Odontoceti) have relatively small brains and cerebella compared to those of the odontocete family Delphinidae (marine dolphins). Dive times of non-dolphin odontocetes have not been compared to those of dolphins of similar body sizes. Furthermore, potential associations between maximum dive duration, cerebellum size, and cortical neuron densities have yet to be explored. Researchers previously demonstrated that dolphin brains had higher cortical neuron densities than that of one mature pygmy sperm whale (*Kogia breviceps*), a small odontocete from the family Kogiidae capable of diving for long periods [11]; dolphins perform relatively short duration dives compared to *K*. *breviceps*, which has a maximum dive time of 47 min [12–14]. Researchers have examined the anatomical composition of shallow and deep diving mammals in relation to the differential metabolic costs of body tissues [15]. Their findings suggest that deep divers invest a smaller percentage of total body mass in metabolically expensive brain and viscera, and a larger percentage of body mass in less energetically expensive skin, bone, and muscle.

**Fig 1:**
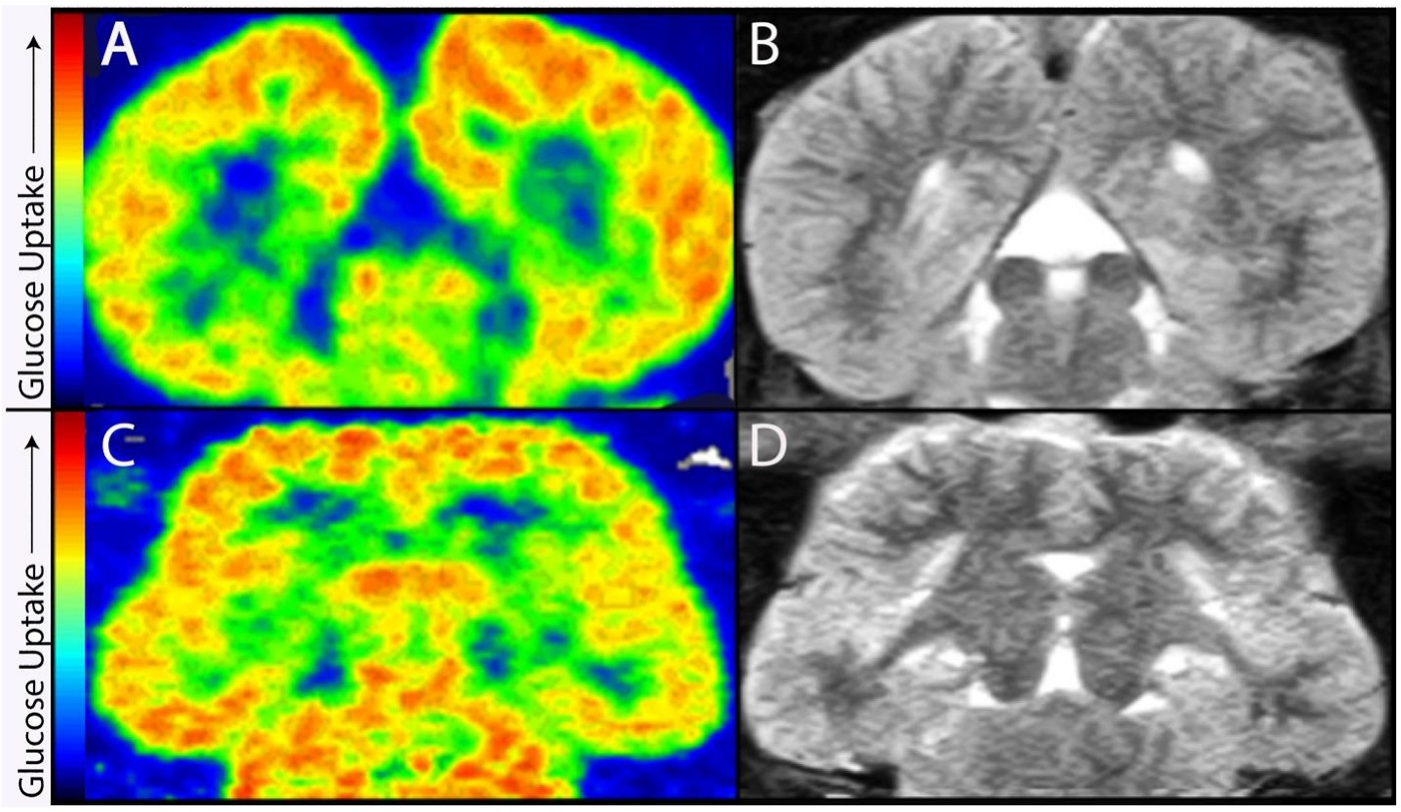
PET scans show that gray matter of the dolphin cerebrum and cerebellum have the highest metabolism in the brain. Frontal (A, B) and horizontal (C, D) views of a *Tursiops truncatus* brain after FDG PET (A, C) and MRI (B, D) scans. Images have been modified from previous publications with the authors’ permission [7,8]. The color map indicates the relative degree of glucose metabolism. The images demonstrate that high metabolic areas (i.e., areas of increased glucose consumption; red) are mainly concentrated in the gray matter of the cerebral cortex and cerebellum, with the exception of smaller sub-cortical nuclei (e.g., inferior colliculi; thalamic gray matter). The PET and MRI scans are from the same healthy dolphin that was trained to lie still in the scanner.

For diving, odontocetes rely upon hearing and sonar to locate prey at depth [16, 17]. As odontocetes search for squid or fish, their muscular nose makes brief trains of echolocation clicks that are focused through a melon-shaped forehead [18–22]. The clicks bounce off the prey, and returning echoes reach the ear (Au 1993), where the cochlea then converts the echoes into nerve impulses [23, 24]. Along axons, the impulses pass from the cochlea to the brainstem and midbrain to reach the gray matter of the cerebral cortex, containing neurons with axonal and dendritic processes along which action potentials are conveyed. The transmission of action potentials along these processes represents a major proportion of the energy budget of the brain [25]. Also, there is evidence that the cerebral and cerebellar cortex of dolphins has the highest metabolism of the brain (Fig 1) [7, 8].

While certain aspects of cortical cytoarchitecture are uniformly present across all mammals, several variations distinguish cetaceans from most mammals. The layering of the cetacean cortex has recently been displayed in great detail [26–28]. For example, cetacean cerebral cortex has a very thick and neuron-sparse layer I, or molecular layer [29–32]. Also, layer IV of the neocortex, which is the primary recipient of sensory information in humans and other primates, is absent across Cetacea. Layer II of the odontocete cortex is characterized by high neuron density and is potentially similar in function to the external granular layer of terrestrial mammals [28]. Clustering of neocortical neurons in layer II of the cortex is present in all odontocetes and in mysticetes, such as humpback and fin whales [26], but not in one mysticete, the bowhead whale [33]. Large cortex surface area is a feature of many cetacean brains [34–36]. The extent of cortical gyrification also varies within Cetacea, with the bowhead whale and some river dolphins displaying less convoluted brains than most other cetaceans [37].

While data on neuronal densities within the neocortex have previously been reported for a limited number of cetacean species and cortical sampling sites [11, 26, 31–33, 38–45], the present study seeks to contribute to and expand upon the existing data to examine potential relationships between neuroanatomical measurements and maximum dive duration in short- and long-diving odontocetes. The data presented include previously reported and original *T*. *truncatus*, *Delphinus delphis* and *Orcinus orca* neuroanatomical measurements [35, 46–48] as well as the first measurements of neuron density from the brains of a neonatal and adult killer whale (*Orcinus orca*) and an adult Cuvier’s beaked whale (*Ziphius cavirostris*), which are rarely available for study. The beaked whale is the longest diver among whales studied to date, with a maximum dive time of 137.5 minutes [49, 50]. Moreover, data from *K*. *breviceps*, another long-diving odontocete, was included in our analysis following the only study of the brain of this species [11]. Here we present observations on brain, cerebral cortex gray matter, and cerebellar mass, CSA, neuronal density, and dive time variability in cetaceans of different taxa. Some researchers have suggested that cetacean cortex is likely to be quite variable across species [26]. We agree with this assessment and were particularly interested in comparing our data on gray matter mass and neuron density with what we know of brain size, cerebellum size, cortex surface area, and maximum dive times of *Z*. *cavirostris* and *O*. *orca*, whales of very similar body size.

## Materials and methods

### Ethics statement

No animals were sacrificed in these studies. All animals died of natural causes and their brains were removed during postmortem examination. The study followed protocols approved by the Institutional Animal Care and Use Committee at the Biosciences Division, Space and Naval Warfare Systems Center (SSC) Pacific and the Navy Bureau of Medicine and Surgery, and followed all applicable U.S. Department of Defense guidelines for the care and use of animals.

The brains examined in the present study came from five odontocete species (*T*. *truncatus*, *D*. *delphis*, *O*. *orca Z*. *cavirostris*, and *Kogia breviceps*; abbreviated as *Tt*, *Oo*, *Zc*, *Kb*, *and Dd*) that died of natural causes in human-managed care or after stranding on beaches. The Zc, Kb, and Dd stranded on beaches in California and were collected by local stranding networks authorized under the United States Marine Mammal Protection Act. The Tt and the Oo were maintained in accordance with regulations under the U. S. Animal Welfare Act. All brains were submitted to us for postmortem analysis. No additional or pre-existing samples were used in this study. The brains were removed at necropsy within 12 hours of the individual’s natural death and were free of neuropathologies. On removal, all brains were immersion-fixed in neutral buffered 10% formalin. Once hardened, the brains were sectioned to measure cerebral cortex surface area (CSA) by using previously published methods [34, 35]. Brain and cerebellum masses and CSA were previously reported for each individual included in this study [37].

**Fig 2:**
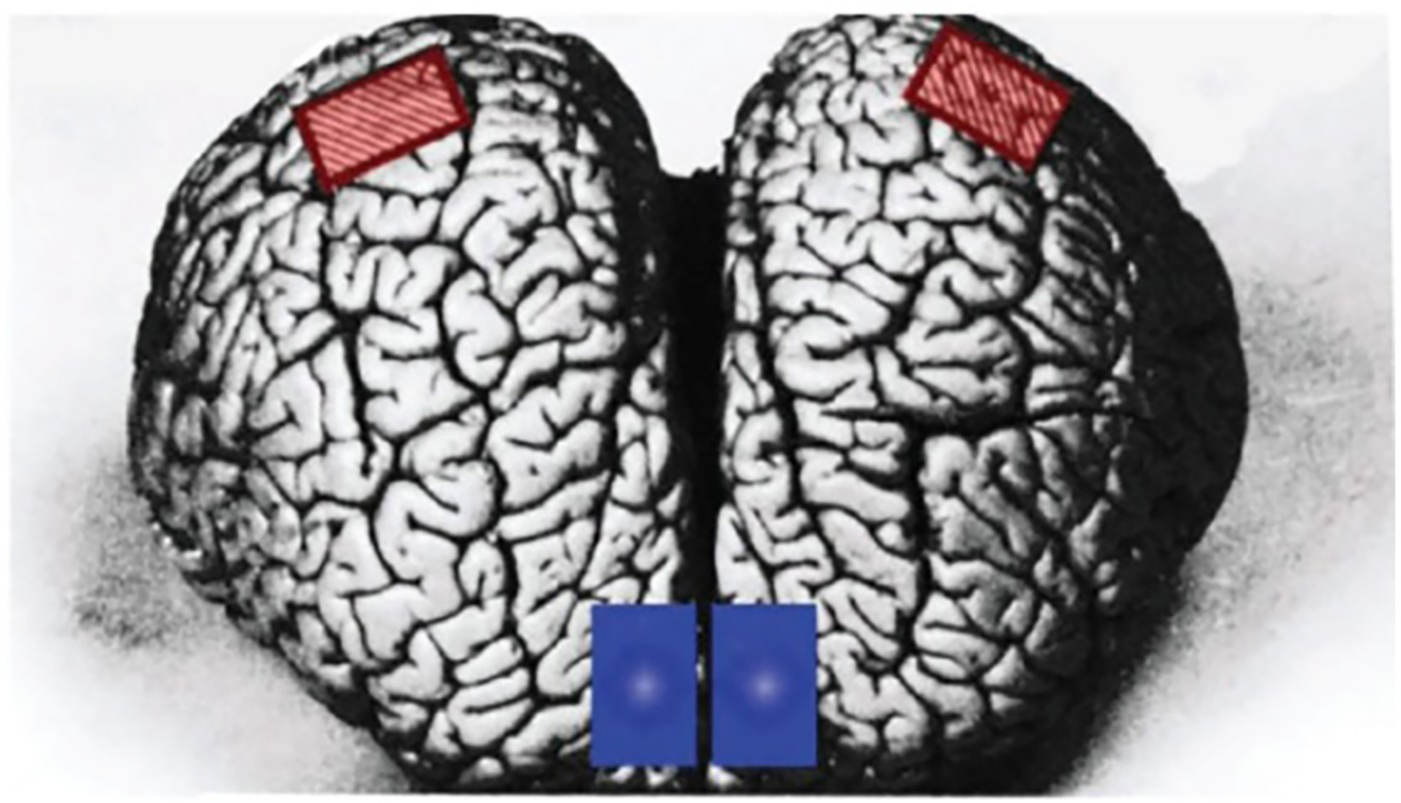
Boxes indicate where the brain samples were taken for the study. Anterior-superior view of the *Tursiops* truncatus (*Tt*) brain. The supralimbic (auditory; red) and anterior paralimbic (motor; blue) areas are marked on either side of the brain.

Tissue specimens were taken from four sites in the cerebral hemispheres, the left and right supralimbic and anterior paralimbic lobules (Fig 2). The fixed specimens were mounted in paraffin, sectioned at 7 microns, stained with cresyl violet (Nissl method), and glial fibrillary acidic protein (GFAP). With light microscopy, photographic montages were prepared from each cortical site from both brain hemispheres, with each section oriented and photographed perpendicular to the pial surface. These images and a grid were projected on a monitor, and magnification and units of measurement were encoded in an image analyzer (Optomax, Hollis, NH, USA). The image analyzer allowed for determination of nuclear area and nuclear diameter of both neurons and glia (Table 1). For all sampling sites in both hemispheres Neuron densities were determined and compared for each cortical layer as well as for the entire cerebral cortex for all areas examined. Glial cells were identified by GFAP staining and their smaller size and their lack of cytoplasm. To calculate cell density, a grid area (0.03 mm^2^) was placed over each cell layer. All neurons and glial cells were recorded in each layer from the pial surface to the white matter border. Converting actual cell counts from counts/mm^2^ to counts/mm^3^ required the use of the following equation:

$$N_{v}=\frac{N}{A(\overset{-}{D}+T)}$$

N_v_ = neurons counted/mm^3^

N = counted cells

A = area counted 0.03 mm^2^

D = mean cell (nuclear) diameter (see Table 1)

T = thickness of tissue sections 0.007 mm

**Table 1:** A trend for larger paralimbic neurons in larger brains.

	Neurons		Neuroglia	
Brain Area	Area	Diameter	Area	Diameter
	*Delphinus delphis* (783)			
Anterior Paralimbic Lobule	89.4	10.7	34.5	6.6
Supralimbic Lobule	76.5	9.9	37.4	6.9
	*Tursiops truncatus* (1,559)			
Anterior Paralimbic Lobule	109.7	11.8	35.8	6.8
Supralimbic Lobule	94.5	11	35.7	6.7
	*Ziphius cavirostris* (2,004)			
Anterior Paralimbic Lobule	107.6	11.7	47	7.7
Supralimbic Lobule	76.5	9.9	39.3	7.1
	*Orcinus orca* adult (6,215)			
Anterior Paralimbic Lobule	130.7	12.9	40.2	7.2
Supralimbic Lobule	77.2	9.9	33.3	6.5
	*Orcinus orca* neonate (3,292)			
Anterior Paralimbic Lobule	112.4	12	--	--
Supralimbic Lobule	83.5	10.3	--	--

Comparison of the mean neuron and glial cell nuclear areas and nuclear diameters in the cerebral cortex of the Odontocete by brain area. Brain size in grams for each species is indicated in parentheses. Consistent with the findings of Haug [36], there was a trend for larger neurons in the larger brains.

Cortical surface area (S) and cortical gray matter thickness (T) were used to calculate the total volume of gray matter (G), G = ST [51]. Gray matter mass was then calculated by multiplying gray matter volume by 1.036 g/cm^3^, the specific gravity of gray matter in humans [52]. We previously found an average value of 1.04 g/cm^3^ for the entire cetacean brain, including white and gray matter [37].

The total number of neurons in the gray matter of the cerebral cortex was calculated for each species from neuron density, cortical surface area (CSA) and thickness (T) measurements [36]. Our total neuron counts for *Tt*, a species whose neurons have been enumerated by several investigators, are within the lower range of published values [31, 32, 36, 40].

The *Kb* was not part of our initial study. Our *Kb* data were derived from Nissl stained sections and an automated counting procedure (Reveal Biosciences, San Diego, CA). Only total counts were done in this species. There were not counts of separate layers.

All statistical comparisons were made using a two tailed T-test.

## Results

### Neuron density and cortical measurements from the brains of five odontocetes

**Fig 3:**
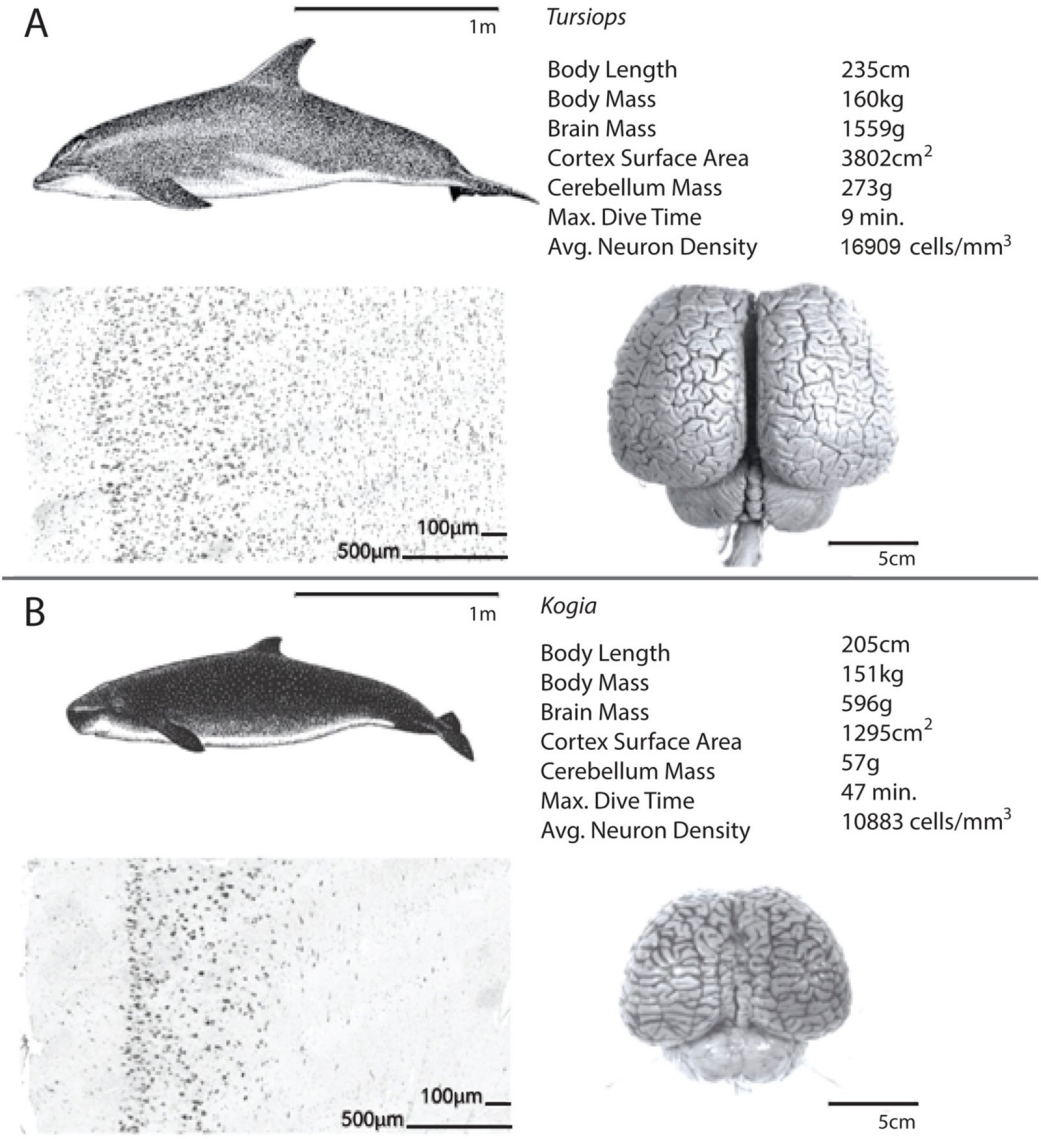
Comparison of a smaller short diver and a smaller long diver. An illustration of the differences in brain mass, cortical surface area, cerebellum mass, average neuron density, and maximum dive time between one female adult delphinid (*Tursiops truncatus*; A) and one female subadult individual from the family Kogiidae (*Kogia breviceps*; B) of similar body size.

**Fig 4:**
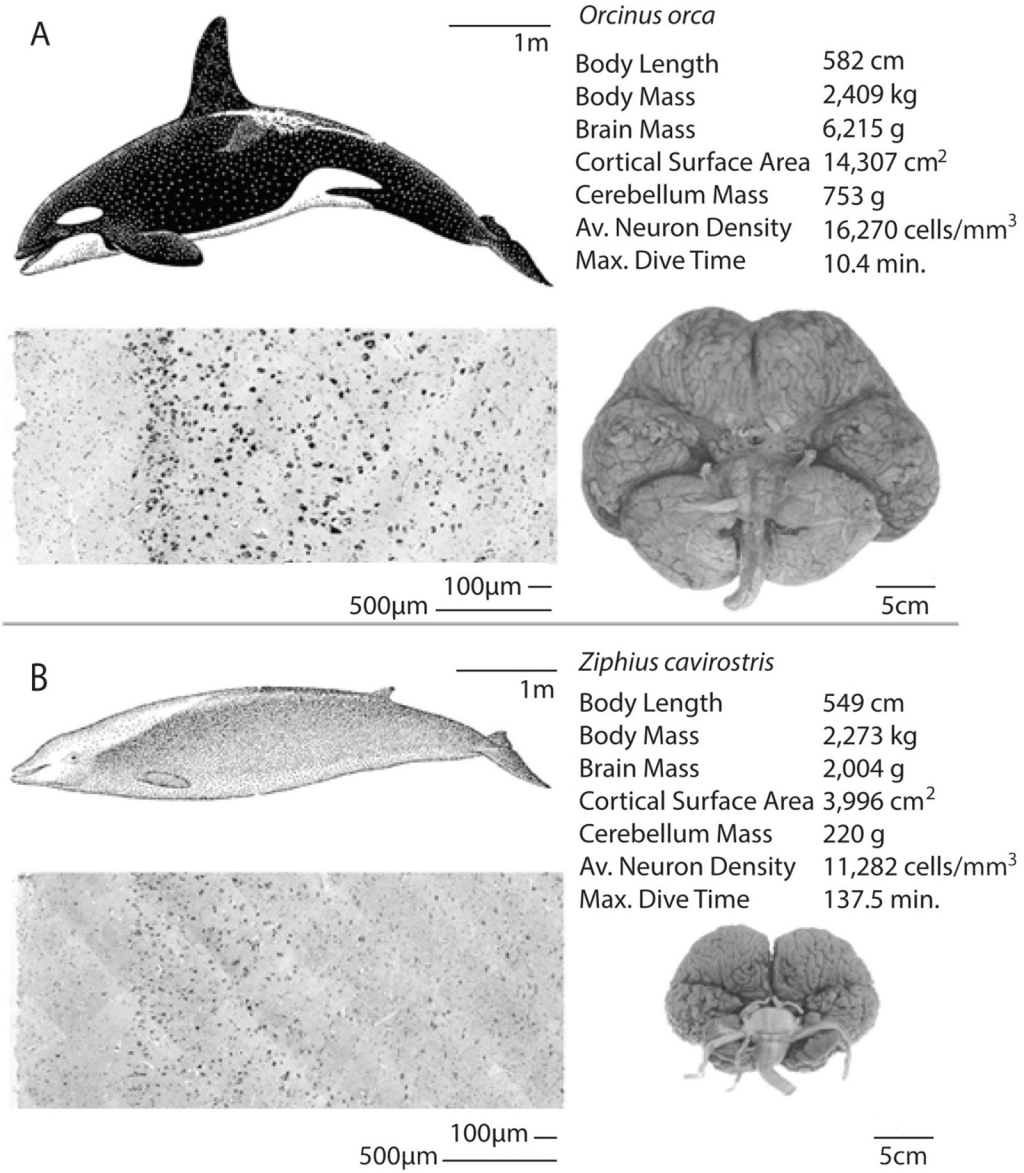
Comparison of a larger short diver and a larger long diver. An illustration of the differences in brain mass, cortical surface area, cerebellum mass, average neuron density, and maximum dive time between one female adult delphinid (*Oo*; A) and one female adult ziphiid (*Zc*; B) of similar body size. *Zc*, which can dive deeper than a mile and longer than an hour, has a smaller brain with fewer neurons than that of *Oo*, an animal of similar body size that performs shallower, shorter dives.

Average neuron densities, cell sizes, and other anatomical data for the five odontocete species examined in the current study are presented in Tables 1–3 along with data from other species reported in separate studies in S1 Table. We compare our data from adult odontocetes of similar body size (*T*t and *Kb* and *Oo* and *Zc* all females) in Figs 3 and 4. These two pairs of species differ greatly in diving capability despite body size similarities. The maximum dive time of *Zc* (>2 hr) is over 12 times longer than that of *Oo*. Kb dives more than five times longer than Tt. Both pairs of females differ in whole brain, cortical gray matter, and cerebellum mass, cortical surface area, and neuronal density (Figs 3 and 4).

**Table 2:** Neuron density by cortex layer.

Brain Area	Cell Layer	Neurons						
		I	II	IIIA	IIIB	V	VI	Area Mean
	*Delphinus delphis (Brain*: *783g)*							
APL		2,970	343,613	18,008	11,630	14,256	10,598	16,846
SLL		2,814	57,963	30,201	24,386	17,914	16,038	24,886
Layer Mean		2,892	50,788	24,105	18,008	16,085	13,318	**20,866**
	*Tursiops truncatus (Brain*:*1*,*559g*)							
APL		4,690	35,224	13,765	10,630	12,922	11,463	14,782
SLL		1,876	43,144	21,728	17,508	15,632	14,325	19,036
Layer Mean		3,283	39,184	17,747	14,069	14,277	12,894	**16,909**
	*Ziphius cavirostris (Brain*: *2*,*004g*)							
APL		938	23,448	9,379	8,910	9,848	10,786	10,552
SLL		321	34,234	11,724	7,503	12,193	6,096	12,012
Layer Mean		630	28,841	10,552	8,207	11,021	8,441	**11,282**
	*Orcinus orca (Brain*: *6*,*215g*)							
APL		285	31,889	13,131	10,183	11,255	7,503	12,374
SLL		703	42,441	22,510	23,213	16,648	15,476	20,165
Layer Mean		494	37,165	17,821	16,698	13,952	11,490	**16,270**

Neuron cell packing density ranges for four species of odontocetes by cell layer and brain area. APL = anterior paralimbic lobule; SLL = supralimbic lobule. All values represent average neuron counts.

**Table 3:** Glia density by cortex layer.

Brain Area	Cell Layer	Neuroglia						
		I	II	IIIA	IIIB	V	VI	Area Mean
	*Delphinus delphis (Brain*: *783g)*							
APL		48,944	36,733	39,215	47,852	47,852	48,149	44,791
SLL		40,902	37,130	33,357	36,633	41,796	40,009	38,305
Layer Mean		44,923	36,932	36,286	42,243	44,824	44,079	**41,548**
	*Tursiops truncatus (Brain*: *1*,*559g*)							
APL		26,859	32,274	30,108	36,390	28,808	36,173	31,769
SLL		32,991	29,875	32,807	34,274	41,422	36,473	34,640
Layer Mean		29,925	31,075	31,458	35,332	35,115	36,323	**33,205**
	*Ziphius cavirostris (Brain*: *2*,*004g*)							
APL		35,144	32,166	36,931	29,783	43,483	47,057	37,427
SLL		28,592	22,040	35,144	44,079	33,357	42,292	34,251
Layer Mean		31,868	27,103	36,038	36,931	38,420	44,675	**35,839**
	*Orcinus orca (Brain*: *6*,*215g*)							
APL		29,188	29,485	25,018	21,146	22,933	22,933	25,117
SLL		34,846	26,507	27,401	28,592	30,677	29,783	29,634
Layer Mean		32,017	27,996	26,210	24,869	26,805	26,358	**27,376**

Glial cell packing density ranges for four species of odontocetes by cell layer and brain area. APL = anterior paralimbic lobule; SLL = supralimbic lobule. All values represent average glial cell counts.

Consistent with all previous studies [26–28, 31, 40, 53], the present study of the cerebral cortex of five odontocetes found a thick neuron sparse layer I, a lack of layer IV as well as a high density of neurons in layer II within the four cortical areas sampled (Fig 2). Although one study [40] noted a trace of layer IV in a *Tt* neonate, the current results from the *Oo* neonate did not reveal a trace of layer IV; however, the authors of this earlier study sampled slightly different areas of cortex compared to our own samples. Neuron densities comparisons between left and right cerebral hemispheres (Fig 2), revealed no significant differences in the areas sampled (*P* = 0.03) for all species. Previously, we found a slight but significant right hemisphere advantage in cortical surface area for *Tt* and *Dd* [54]. In a follow-up study including the same two species, we found no significant difference in cortical thickness between the two hemispheres [46]. Layer 1, or the molecular layer [30, 36, 55], was relatively thick in all species that we studied but layer 1 in our specimens were all sparse in neurons and contained numerous glia.

In agreement with other published work [28, 44], we found cortical thickness differences across the cetacean brains studied. The cortical thickness of the *Oo*, which has the largest body and brain mass within the family Delphinidae, was greater than that of the other delphinids (*Tt* and *Dd*) examined. However, the cortical thicknesses of *Zc* and *Oo* were very similar, despite a three-fold difference in total brain mass (Fig 4). We found that *Tt* and *Dd* cortical thicknesses did not differ significantly, as was found by another previous study [53].

### Cerebellum comparisons

Compared to *Kb* and *Zc*, delphinids (*Oo*, *Tt*, and *Dd*) have a larger proportion of the brain devoted to the cerebellum [37, 56](Figs 3 and 4). The amount of cerebellum relative to total brain mass in *Zc* and *Kb* (11% and 10%, respectively) is similar to that of humans [57]. Relative to their body size, delphinids have the largest cerebella compared to other cetaceans [37] and other large terrestrial mammals, including elephants [57]. Humans and other mammals have more neurons in the cerebellum than in the cerebral cortex [57]. Although delphinids have high neuronal density within the cerebellum, it is less than that of humans according to another study [58], which reported neuron densities of 572 cells/per 0.001 mm3 in the *Tt* cerebellum compared to 721 cells/per 0.001 mm3 in the human cerebellum. As the cerebellum of *Tt* is about 53% larger than that of humans [59], this suggests that *Tt* has about over 20% more total neurons within its cerebellum than humans. In *Tt* there is also support for high metabolism and circulation in the living dolphin cerebellum [8]. In addition to the high metabolism suggested by ^18^F-2-fluoro-2-deoxyglucose positron emission tomography (FDG PET) scans in the living dolphin [7], another PET study observed rapid cerebellar and cerebral cortex uptake using short half-life radiolabeled ammonia 13NH^3^ [8].

### Neonatal comparisons

**Fig 5:**
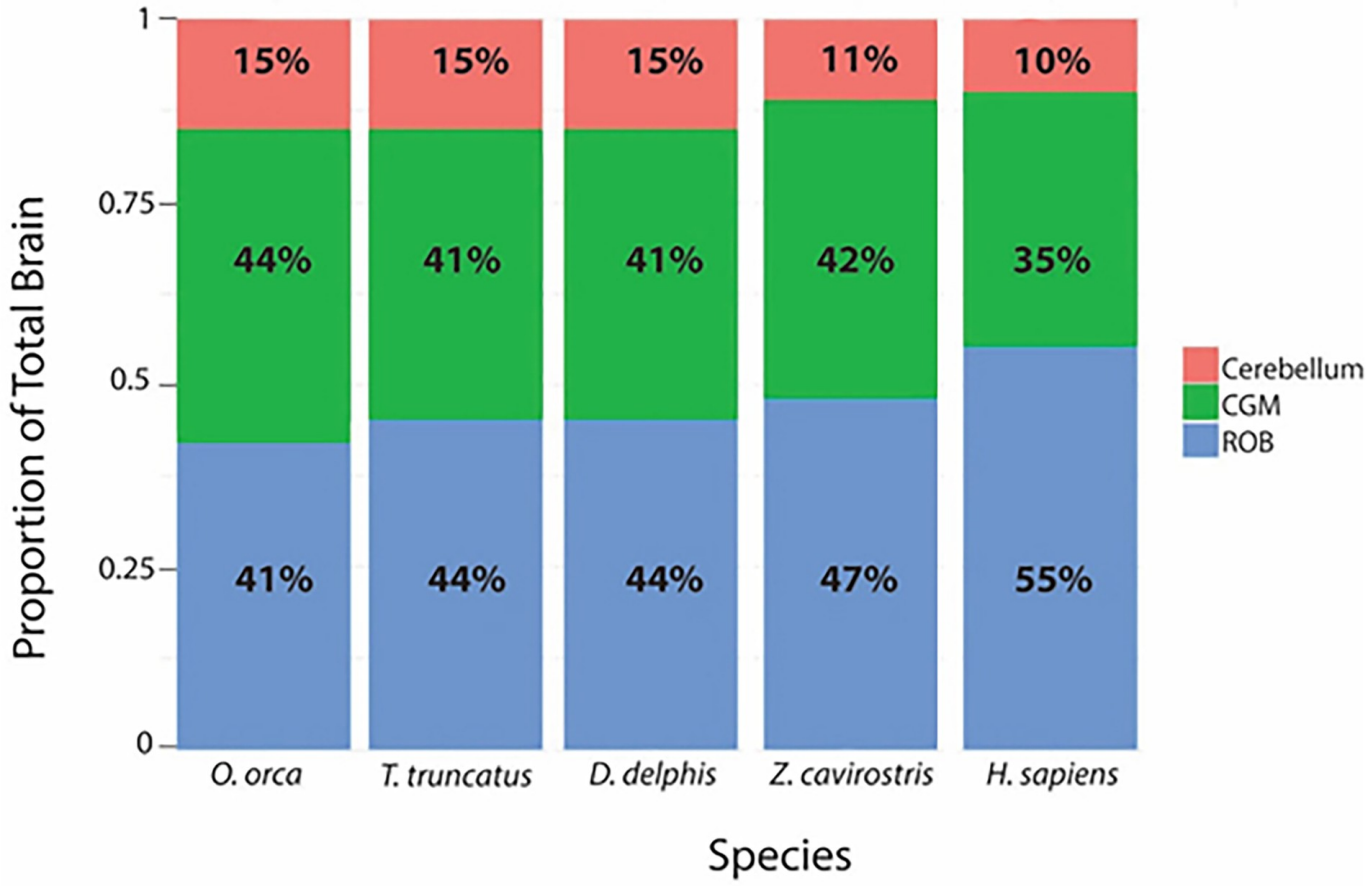
Comparison of neonates and adults for the only two cetacean species where such data is available. Brain and body size, brain cell density, and cortical surface area comparisons between neonates and adults representing two dolphin species, *Tt* (A) and *Oo* (B). All data are from individual animals. Brain and body mass data as well as neuron and glial cell densities for the neonate *Tt* were published previously [40].

With the addition of data from our neonatal *Oo* specimen to the literature, there are now neuron and glial cell packing density measurements for neonatal cetaceans of two species, *Tt* and *Oo*. Fig 5 compares the neuron and glial cell densities, brain, cerebellum, and cortical gray matter masses, and cortical surface areas of *Tt* and *Oo* neonates and adults. The neonate *Tt*, described in a previous study [40], had the highest neuron and glial cell densities overall, at 48,700 cells/mm^3^ and 77,300 cells/mm^3^, respectively. The mature *Tt* brain was about 2.5 times larger in mass than the neonatal *Tt* brain and the cortical neuron density of the neonatal *Tt* brain was about 2.5 times greater than that of the mature *Tt* brain.

The neonatal *Oo*, with a much larger body size and brain almost five-fold larger than that of the neonatal *Tt* [40] had neuron and glial cell densities that were around half those of the *Tt* neonate (*Oo* neonate neuron density: 21,503 cells/mm^3^; glial cell density: 37,030 cells/mm^3^). However, despite the great differences in brain size and brain cell density between these two delphinid species, the ratios of glia to neurons were similar (adult *Tursiops*: 1.95, neonate *Tursiops*: 1.59, adult *Orcinus*: 1.32, neonate *Orcinus*: 1.72). Both neuron and glial cell densities were higher in neonates compared to mature adults.

Glial cell density disparities between neonate and adult delphinids were comparable to those in neuron density. The brain mass of the adult *Oo* was approximately twice that of the neonate *Oo*. Similarly, the glial cell density of the neonate *Oo* cerebral cortex was nearly twice that of the mature *Oo*. However, the cortical neuron density of the neonate *Oo* was only 1.4 times greater than that of the mature *Oo*.

## Discussion

### Brain size, neuron and glial density and dive time

For the present study, we primarily focus on the relationships between brain-body measurements, neuronal densities, and dive time. Our neuron density measurements add to the very sparse literature on these values in cetacean brains. Of particular interest was the relationship between neuron densities with brain size. Previous studies [38, 39] have suggested that neuronal density is a function of brain size rather than taxonomic relationships. The glia/neuron ratio rises from small-brained rodents (mouse, rabbit: 0.35) to ungulates (pig, cow, and horse: 1.1), humans (1.68–1.78), and *Tt* (1.95) of intermediate brain size, to large-brained whales (fin whale: 4.54–5.85) [39, 60].

One published review of the literature [41] suggests that cetaceans have relatively low neuron densities and high ratios of glia to neurons. In contrast to early studies [38, 39], more recent studies suggest that taxonomic as well as brain size differences may affect neuron density in cetaceans [11, 43]. Even earlier, one study [36] noted “The density of glial cells in the gray of cortex shows very large variations. A dependence on brain size cannot be observed.” One published report [61] demonstrated that *Tt* had neuron densities as high as *P*. *phocoena*, a species with a brain only one-third as large as that of *Tt*. Furthermore, two species of river dolphins (*Platanista*) with brains only one-seventh as large as *Tt* had similar neuron densities to *Tt* [61].

Previous studies [38, 39] have suggested that neuron density was a function of brain size rather than taxonomy. Instead, we suggest that neuron density likely follows a particular trend based on species taxonomy and family-specific features, such as metabolism, gestation duration, and ecology [37]. Some cetacean species, such as *D*. *leucas* and *Z*. *cavirostris*, display similar neuron densities and brain masses despite significant differences in body size.

Across species and compared to neonates of the same species, adult body size is vastly different, thus encephalization quotient (EQ) is also quite different. EQ is not a good indicator of neuron density. Again, there seems to be a trend in neuron density based on taxonomic family. Also, *Dd*, *Tt*, *Oo* are in the same dolphin family and their average neuron density decreases with increasing brain and cerebral cortex size in mature animals. Previous studies in land mammals have presented the scaling relationship between neuronal density and brain size as being order-specific [5, 60]. Although the present study only presents data for a limited number of species within non-delphinid cetacean families, these data may suggest family-specific scaling patterns for neuronal density in cetaceans. This is suggested by our comparisons of neuroanatomical data from *Tt*, a delphinid, with *Kb*, a kogiid [11], which supports a previous study demonstrating that neuron density is much lower in *Kb*, compared to *Tt*, despite its much smaller brain [11].

Also, when we compare delphinid *Oo* with ziphiid *Zc* two animals of very similar body size, we find that *Oo* has a higher neuron density despite having a brain three times as large as that of *Zc*. However, within their taxonomic group, our results show that in family Delphinidae, cortical neuron density decreases with brains enlargement from *Dd* to *Tt* to *Oo*. Taken together, the aforementioned comparisons indicate that body and brain size are not in every case the determining factors of cortical neuron density.

Among mammals, the glia/neuron ratio is species-specific, and the number of glial cells varies with the number of neurons during ontogenesis [43]. By comparing multiple brain measurements in available brains from short-diving delphinids with brains from two non-delphinid odontocetes known for longer dive durations, the present study extends these results.

One study [62] found a higher ratio of glia to neurons in the human frontal cortex compared to other primates with much smaller brains. They suggested that “relatively greater numbers of glia in the human neocortex relate to the energetic costs of maintaining larger dendritic arbors and long-range projecting axons in the context of a large brain.” The neuron-glia relationship may be somewhat different in cetaceans. For example, the neonatal *Oo*, with a much larger body size and brain over five times larger than that of the neonatal *Tt*, had brain cell densities (neuron density: 21,503 cells/mm^3^; glial cell density: 37,030 cells/mm^3^) that were less than half of those of the *Tt*. However, glia/neuron ratios for neonate *Tursiops* and *Orcinus* are quite similar (*Tursiops*: 1.59, *Orcinus*: 1.72). The glia/neuron ratio in the deep- and long-diving *Zc* (3.18) with a brain of 2004 g is twice that of the shallow- and short-diving *Oo (1*.*68)* with a brain of 6215g (S1 Table). Having higher glia/neuron ratios while performing long, deep dives into cold and dark ocean waters with limited oxygen stores may facilitate heat production [41]. In addition, higher glia/neuron ratios may enhance neurotransmission of acoustic information or protect the brain from hypoxia at depth. For example, glia can serve as a sink for carbon dioxide during periods of hypoxia [63].

### Dive capabilities and the cerebellum

There is a dichotomy in cerebellum size between the delphinoids (Phocoenidae, Monodontidae, and Delphinidae) and other odontocetes. After controlling for brain size, the average delphinid cerebellum is 17.2% larger than the average ape cerebellum and 53.5% larger than the average human cerebellum [64]. However, there is great diversity in relative cerebellum size across cetaceans. For example, the largest delphinid, *Oo*, has a cerebellar quotient (CQ) about twice as high as that of the giant sperm whale (*Physeter macrocephalus*) [65], and about 70% higher than that of *Zc* (*Oo*: 753 g cerebellum [CQ = 1.36]; *Zc*: 206 g cerebellum [CQ = 0.8]). Both *P*. *macrocephalus* and *Zc* can dive over six times longer (one to two hours) than *Oo* (just over 10 minutes). *Zc* has a smaller brain and a lower neuron density compared to *Oo*, resulting in a comparatively lower total neuron count (Fig 4, Table 2). Although other anatomical and physiological features contribute to the ability of ziphiids and kogiids to perform longer and deeper dives [15, 66], lower brain metabolism due to a smaller whole brain and cerebellum relative to body size with fewer neurons in cortex gray matter is likely another feature that may be an advantage in diving. This comparison of odontocetes appears to show that lower total neuron counts and neuronal densities, and smaller cerebella are all correlated with longer dive times (Figs 3 and 4).

One study of terrestrial mammals [5] notes that total glucose use by the whole brain, cerebral cortex, or cerebellum is directly related to the number of neurons within each structure. A higher total neuron number yields a higher energy budget for the brain as a whole. This increased energy budget may actually reflect the relatively large amount of mitochondria that is present in gray matter (particularly in dendrites and axon terminals) compared to white matter [67]. Thus, the amount of mitochondria may be the mediating factor in the negative correlation between relative gray matter mass and dive time. Having a relatively low number of neurons and a lower brain energy budget may facilitate the longer, deeper dives of some cetaceans.

Dive time in cetaceans may also depend on the efficiency of oxygen use in muscle tissue and in the bloodstream. How much muscle and blood oxygen reserves a cetacean has is related to the dive duration and oxygen demands [68]. Cetaceans with higher demands for oxygen have increased oxygen reserves compared to those that typically dive for shorter periods of time.

In the non-cetacean mammals investigated so far, the greatest neuron density and the largest total number of neurons are found in the cerebellum [57]. Relative to body size, the cerebellum of members of the family Delphinidae is the largest among Cetacea and possibly of all mammals. The bottlenose dolphin (*Tt*) cerebellum is over 50% larger than that of humans [57, 59], yet its cerebellar neuron density is only slightly less than that in humans [58]. Considering the cerebellum size and neuron density data reported [58], the total neuron cell count of the bottlenose dolphin cerebellum is approximately 20% greater than that of humans.

### Total numbers of neurons in the cerebral cortex and cerebellum

Recent work [5, 69] suggests that the absolute number of neurons within the cerebral cortex may be a better determinant of cognitive performance than its relative size. Previous studies have reported total neuron counts for additional cetacean species, such as the long-finned pilot whale (*Globicephala melas*), which has a relatively high total neuron count that is nearly twice that of humans [45]. S1 Table demonstrates that delphinids generally have high total neuron counts that are near or (in the case of pilot and killer whales) greater than the total number of neurons in humans. Some human studies [70, 71] have posited that the cerebellum is responsible for more than just movement and motor control, but also for cognitive tasks, including attention, executive control, language, working memory, learning, pain, and emotion. The large number of neurons in the dolphin cerebellum [58] may also account for some of the intricate sensory and cognitive behavior of delphinids.

### Metabolism of the cerebral cortex and sociality

Fig 1 shows horizontal and frontal sections of a living *Tt* brain during positron emission tomography (PET) after uptake of ^18^F-2-fluoro-2- deoxyglucose (FDG) to measure relative brain metabolism from glucose uptake [7]. Magnetic resonance images are shown for anatomical comparison. The images demonstrate that high metabolic areas are mainly concentrated in the gray matter of the cerebral cortex and cerebellum, with smaller areas of high metabolism in the inferior colliculus and thalamic gray matter. Both PET and MRI scans are from the same healthy dolphin that was trained to lie still in the scanners. These particular scans cannot be compared directly with other Tt or cetacean brains since direct comparisons of different brains require PET ligand dosages calibrated for body size. However, these images demonstrate that in *Tt*, as in humans and other mammals, the highest metabolism is in the gray matter.

It is metabolically expensive to have more gray matter, more neurons, and a larger cerebellum. If doing so may also limit dolphins to shorter and shallower dives, what is the advantage that outweighs these limitations? Some proponents of the Social Brain Hypothesis posit that large, metabolically expensive brains support an enhanced social repertoire, cooperation, and prey diversity [6, 72]. This is often taken to mean that large-brained animals can support a large social group. One study indicates that this trend may not be as linear as previously expected among cetaceans [72]; this study found that cetaceans in the largest social groups (e.g. *Dd*), deemed ‘megapods,’ have smaller brains compared to those belonging to mid-sized social groups (e.g. *Tt* and *Oo*). Furthermore, cetaceans living as relatively solitary (e.g. *Zc* and *Kb*) have the smallest brains relative to body size. Our data on neuron density resemble this trend with regard to social group size with one exception. *Dd* have relatively large brains and high neuron densities despite usually belonging to ‘megapods.’ While it is true that cetaceans in mid-sized groups tend to have the richest social repertoires [72], it may also be the case that having greater neuron densities may support complex and expanded social connections, regardless of group size. However, we cannot be certain that neurons of different species are comparable. Our results suggest neurons are larger in the larger brains. One study showed that in fact, the neurons found in cetacean cortex appear to be less complex than those found in the closely related Artiodactyls, having lower overall dendritic lengths, segment lengths, spine numbers, and spine density [73] There were differences in fixation between the different brains in the study. Differences in fixation could amplify neuron differences shown in the study. Still, until equally well fixed material is available, we must consider that neurons differences observed could possibly mean that there are different information processing abilities of neurons of the compared species. In this sense, the social relations mentioned above must be viewed with caution. Future studies may address the question: How can neurons be compared across species?

### Comparison of cortical gray matter mass and neuron density

**Fig 6:**
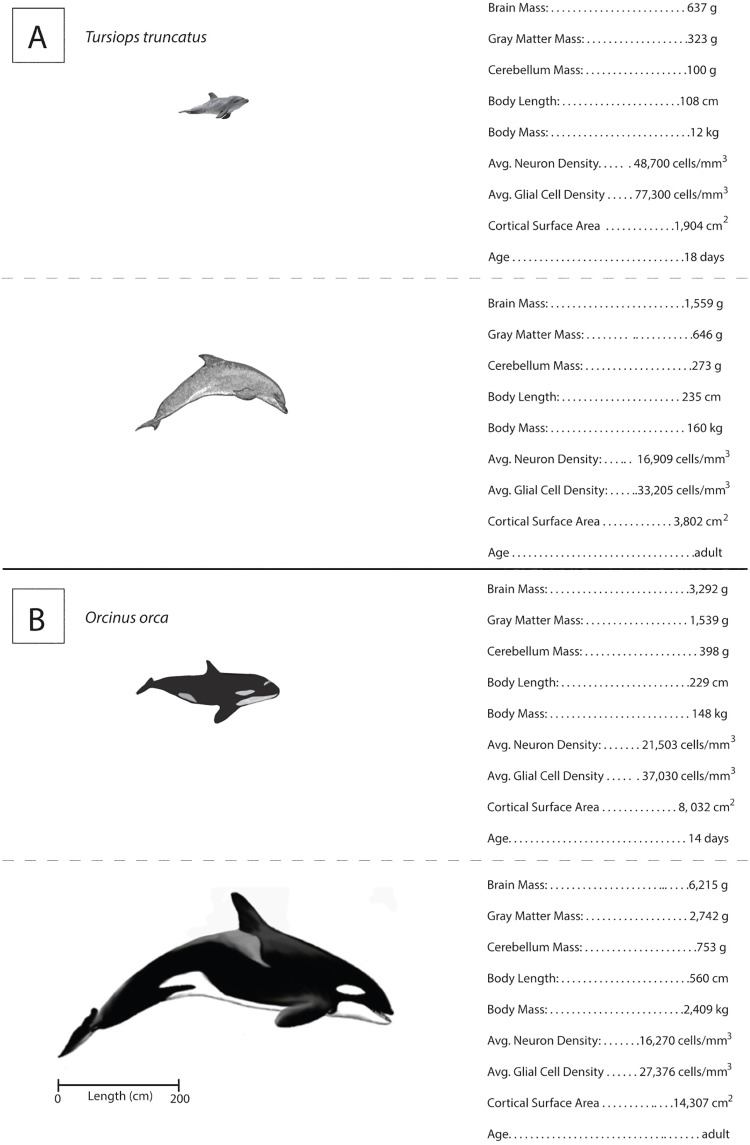
Distribution of total brain mass for mature *Oo*, *Tt*, *Dd*, *Zc*, and *H*. *sapiens*. Each bar represents the percentage of total brain mass in the cerebellum (pink), gray matter of the cerebral cortex (green), and the remaining areas of the brain (ROB: brainstem, white matter of the cerebral cortex) are shown in blue. Mass of the gray matter was calculated by multiplying the cortex surface area and cortex thickness (see S1 Table) by the specific gravity of the gray matter of the cerebral cortex [52]. For average cortex thickness and surface area we used our own measurements. For the human cortex thickness, we used a value of 2.5 mm. The calculated values here are similar to *H*. *sapiens* values averaged from previously published data [74]. Cerebellum masses are values from a previous publication [37]. *Kb* was not included because we only had a brain from an immature animal.

Of the species we studied, *Oo* has the largest percentage of cortical gray matter mass, and humans have the greatest percentage of brain mass in the remaining areas of the brain (ROB), which includes white matter and the brain stem. The percentage of brain mass in the human cerebellum is about equal to that in *Zc* and similar to that in the non-delphinoid odontocetes, such as *Kb* [37]. An even smaller relative cerebellum size is found in *P*. *macrocephalus* and the Indian River dolphin, (*Platanista gangetica*) [37]. Humans have a lower percentage of gray matter mass [72] compared to the cetaceans we studied (Fig 6). Considering the cerebrum alone, the human cerebrum is closer to 60% white matter and 40% gray matter [74, 75].

The gray matter of the human cerebral cortex contains a total of 15 to 20 billion neurons [57, 76, 77]. Although it has been suggested that the human cerebral cortex has the highest total number of neurons compared to that of any other species [67], our findings and those of other investigators suggest that this is not the case. For instance, one study [45] reports a much higher total neuron count for the pilot whale compared to humans (S1 Table). Also, based on our calculations using cortical thickness, cortical surface area, and neuron density, *Oo* specimens have total neocortical neuron counts higher than those of any other species, including *G*. *melas* and humans (S1 Table). *Oo* also has a much larger cortical gray matter mass compared to other cetaceans. It appears that the proportion of the brain occupied by cortical gray matter of the adult female killer whale examined here (44%) is similar to that previously reported for a male killer whale (48%) based on MRI scans of a brain of very similar size (6215g for current specimen and 6435g for another specimen in [56]).

Consistent with a magnetic resonance imaging (MRI) study of the *Oo* brain [56], we found cortical gray matter to be 44% of brain mass in *Oo*, despite its large brain size. Gray matter accounts for approximately 41% of total brain mass in *Dd*, *Tt* and 42% in *Zc* (Fig 5). The comparison of gray matter percentage between dolphins and humans is reminiscent of the comparison of humans with higher primates. The other primates have a higher percentage of gray matter compared to humans [57]. Some observations have emphasized the higher percentage of white matter (axons) in the human frontal cortex [62]. The human advantage in information processing may relate to the more extensive white matter of the frontal cortex.

**Fig 7:**
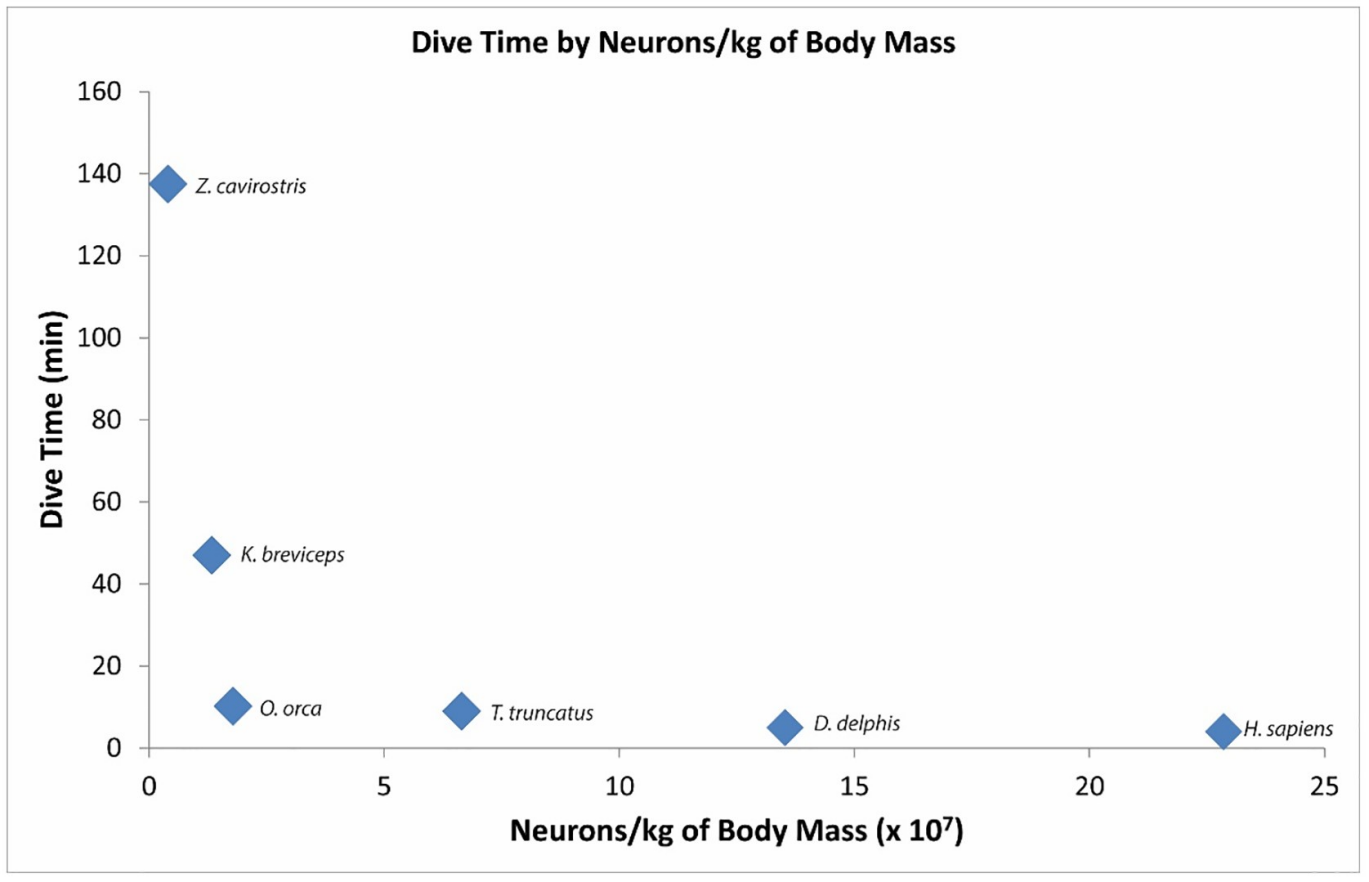
Dive time by neurons/kg of body mass. Maximum dive time by total number of neurons in the cerebral cortex per kilogram of body mass in three delphinids, one member of family Ziphiidae, one member of family Kogiidae, and humans. Dive time data come from previous publications [12, 50, 78–80].

Taken together, these findings demonstrate that brain size alone does not always accurately predict neuron density. In odontocete cetaceans, evolutionary history and taxonomic relationships may play a more important role than brain size in determining neuron density. Furthermore, it appears that neuron density and maximum dive time may also be related in odontocetes, with lower neuron densities and total neuron counts relative to body size appearing to correlate with longer dive times when animals of similar body size are compared (Fig 7).

## Limitations

We have presented our bihemispheric measurements of cortical neuronal density from five cetacean species (*Oo*, *Zc*, *Kb*, *Tt*, and *Dd*) with brains representing almost an order of magnitude difference in size. Cetacean data is largely underrepresented in the literature. The present study began in the 1980s [35, 46, 47, 48] and though limited, provides more samples than those previously published on these different odontocetes. We present the only neuron and glia density data comparing *Oo* and *Zc*. Also, we present the only data comparing neonate and adult killer whales (Oo). The neuron and glia counts were done in the mid-1980s and were presented at conferences with published abstracts only [47, 48]. Other cetacean neuron density data come from a variety of studies. Most cell density estimates are based on samples from different areas of the cerebral cortex. Although our methods for assessment of neuron density could be questioned, we used the same methods and same areas of cortex for all species except for *kb*.

Traditional methods for calculating brain cell density involve stereology, in which cross-sections of brain tissue from various regions of the brain are examined. This is the method employed in the present study. One study [81] described an “alternative non-stereological method, the isotropic fractionator, which involves homogenizing brain tissue into a suspension of countable neuronal and non-neuronal cell nuclei.” Two counting methods, manual and automated, may be used with the isotropic fractionator [82]. One publication [83] warned of the dangers of under sampling with stereological methods. For example, another study [84] reported a rather extreme neuron count of 14.9 billion neurons in the cerebral cortex of the small harbor porpoise (*Phocoena phocoena*), which is close to the total cortical neuron count for humans. It has been posited that this extreme value was likely due to an invalid extrapolation after sampling too few cells within cortical sections [82]. However, researchers have compared the stereology and isotropic fractionator techniques and found no consistent or statistically significant differences in the results obtained from both methods when sufficient samples were taken [82].

The same conclusions were drawn from a study using two different species (humans and macaques) [85]. Furthermore, another study [86] found that the relationship between average estimates and the variance of estimates for a given tissue sample was comparable across all techniques (manual and automated counting with the isotropic fractionator, and stereology). The main advantage of using the isotropic fractionator is faster processing time, whereas the key disadvantage is destruction of the analyzed tissue sample.

As mentioned above, methods vary. Going forward, more standardized methods for calculating cell density must be established in order to accurately compare data across individuals, developmental stages, and species. Only a few brains from a limited number of cetaceans have been studied. To our knowledge, only two neonatal cetacean brains have been studied, comprising one *Tt* neonate [40] and our present study of the *Oo* neonate. We were able to study a single *Zc* that beached alive near our laboratory. Every year many cetaceans live strand on beaches around the world. Given the necessary resources, scientists should be able to examine a wider sample of cetacean brains to fill in the giant gaps in our knowledge. New imaging technology and software for automated cell counting may make it possible to analyze neuron and glia density and other anatomical features such as the microcirculation across the entire brain. Our current findings are a small step in that direction.

## Supporting information

S1 Table Values from the current studies compared to published values. Brain cell densities, cortical surface area and thickness, total number of cortical neurons, and brain and body mass measurements for ten species of cetaceans and humans. Total cortical neuron counts were estimated using our measurements for cortical thickness, average cortical surface area, and average neuron density from each species, with the exception of the total cortical neuron counts for *Gm* and *Hs*. ND_AVG_ = average neuron density of the neocortex (cells/mm^3^); GD_AVG_ = average glial cell density of the neocortex (cells/mm^3^); SA_Cx_ = cortical surface area (cm^2^); T_Cx_ = cortical thickness (mm); N_TOTAL_ = total number of neurons in the neocortex (×10^9^); M_Brain_ = brain mass (g); M_CGM_ = cortical gray matter mass (g); M_Cb_ = cerebellum mass (g); M_Body_ = body mass (kg); * = neonatal specimens; as = anterior supracallosal sector; ps = posterior supracallosal sector; ^▲^ = cortical surface area estimates based on other previously measured brains of similar mass [37]; ^AVG^ = averaged adult data from a previous publication [37]; Family Legend: Bal. = Balaenopteridae; Mon. = Monodontidae; Phoc. = Phocoenidae; Ziph. = Ziphiidae; Kog. = Kogiidae; Delph. = Delphinidae; Hom. = Hominidae. Species legend: *Bp* = *Balaenoptera physalus; Mn = Megaptera novaeangliae; Dl = Delphinapterus leucas; Pp = Phocoena phocoena* [87]; *Zc = Ziphius cavirostris; Kb = Kogia breviceps; Pc = Pseudorca crassidens; Dd = Delphinus delphis; Tt = Tursiops truncatus; Oo = Orcinus orca; Gm = Globicephala melas; Hs = Homo sapiens* [88]. Source legend: auth = measurements by the authors of this study others cited by number in the reference list.
(DOCX)
