# Supplementary material for: Higher neuron densities in the cerebral cortex and larger cerebellums may limit dive times of delphinids compared to deep-diving toothed whales
Source: PLoS One. 2019 Dec 16;14(12):e0226206. doi: 10.1371/journal.pone.0226206 (PMC6914331; doi:10.1371/journal.pone.0226206)
Supplement: S1 Table — Brain cell densities, cortical surface area and thickness, total number of cortical neurons, and brain and body mass measurements for ten species of cetaceans and humans. Total cortical neuron counts were estimated using our measurements for cortical thickness, average cortical surface area, and average neuron density from each species, with the exception of the total cortical neuron counts for Gm and Hs. NDAVG = average neuron density of the neocortex (cells/mm3); GDAVG = average glial cell density of the neocortex (cells/mm3); SACx = cortical surface area (cm2); TCx = cortical thickness (mm); NTOTAL = total number of neurons in the neocortex (×109); MBrain = brain mass (g); MCGM = cortical gray matter mass (g); MCb = cerebellum mass (g); MBody = body mass (kg); * = neonatal specimens; as = anterior supracallosal sector; ps = posterior supracallosal sector; ▲ = cortical surface area estimates based on other previously measured brains of similar mass [37]; AVG = averaged adult data from a previous publication [37]; Family Legend: Bal. = Balaenopteridae; Mon. = Monodontidae; Phoc. = Phocoenidae; Ziph. = Ziphiidae; Kog. = Kogiidae; Delph. = Delphinidae; Hom. = Hominidae. Species legend: Bp = Balaenoptera physalus; Mn = Megaptera novaeangliae; Dl = Delphinapterus leucas; Pp = Phocoena phocoena [87]; Zc = Ziphius cavirostris; Kb = Kogia breviceps; Pc = Pseudorca crassidens; Dd = Delphinus delphis; Tt = Tursiops truncatus; Oo = Orcinus orca; Gm = Globicephala melas; Hs = Homo sapiens [88]. Source legend: auth = measurements by the authors of this study others cited by number in the reference list. (DOCX) [file pone.0226206.s001.docx]

**S1 Table. Values from the current studies compared to published values.**

| **Family** | **Species** | **ND_AVG_** | **GD_AVG_** | **SA_Cx_** | **T_Cx_** | **N_Total_** | **M_Brain_** | **M_CGM_** | **M_Cb_** | **M_Body_** | **Source** |
| --- | --- | --- | --- | --- | --- | --- | --- | --- | --- | --- | --- |
| Bal. | *Bp* | 6,800 | - | - | - | - | 6,856^AVG^ | - | - | 39,365^AVG^ | [38] |
|  | *Mn* | 8,325 (as) | - | - | 2.08 | - | 5,869^AVG^ | - | - | 36,797^AVG^ | [31] |
|  |  | 8,357 (ps) |  |  |  |  |  |  |  |  |  |
| Mon. | *Dl* | 12,332 | - | - | - | - | 1,505 | - | - | 560^AVG^ | [31] |
| Phoc. | *Pp* | 18,350 | - | - | 1.6 | - | 506^AVG^ | - | - | 57.5^AVG^ | [44] |
|  | *Pp* | 13,223 | - | 1,300 | 1.6 | 2.75 | 500 | 215 | - | - | [36, 87] |
| Ziph. | *Zc* | 11,282 | 35,839 | 3,996 | 2.01 | 9.1 | 2,004 | 832 | 220 | 2,273 | auth |
| Kog. | *Kb* | 10,883 | - | 1,295 | 1.43 | 2.02 | 596 | 188 | 57 | 151 | [37] |
| Delph. | *Pc* | 16,597 | - | 7,392^▲^ | - | - | 4,307 | - | - | 483^AVG^ | [36] |
|  | *Dd* | 20,866 | 41,548 | 1,980 | 1.7 | 6.7 | 783 | 321 | 136 | 75 | auth |
|  | *Tt* | 16,909 | 33,205 | 3,802 | 1.64 | 12.7 | 1,559 | 646 | 273 | 186 | auth |
|  | *Tt* | 23,000 | - | 3,110^▲^ | 1.64 | 11.7 | - | 528 | - | - | [40] |
|  | *Tt** | 48,700 | 77,300 | 1,904^▲^ | 1.64 | 15.2 | 637 | 323 | 100 | 12 | [40] |
|  | *Oo** | 21,503 | 37,030 | 8,032 | 1.85 | 31.9 | 3,292 | 1,539 | 398 | 148 | auth |
|  | *Oo* | 16,270 | 27,376 | 14,307 | 1.85 | 43.1 | 6,215 | 2,742 | 753 | 2,409 | auth |
|  | *Gm* | - | - | 7,503^▲^ | - | 37.2 | 3,499^AVG^ | - | - | 1,369^AVG^ | [45] |
| Hom. | *Hs* | 13,520 | 49,230 | 2,000 | 2.5 | 16.3 | 1,508 | 518 | 154 | 75 | [88] |

Brain cell densities, cortical surface area and thickness, total number of cortical neurons, and brain and body mass measurements for ten species of cetaceans and humans. Total cortical neuron counts were estimated using our measurements for cortical thickness, average cortical surface area, and average neuron density from each species, with the exception of the total cortical neuron counts for *Gm* and *Hs*. ND_AVG_ = average neuron density of the neocortex (cells/mm^3^); GD_AVG_ = average glial cell density of the neocortex (cells/mm^3^); SA_Cx_ = cortical surface area (cm^2^); T_Cx_ = cortical thickness (mm); N_TOTAL_ = total number of neurons in the neocortex (×10^9^); M_Brain_ = brain mass (g); M_CGM_ = cortical gray matter mass (g); M_Cb_ = cerebellum mass (g); M_Body_= body mass (kg); * = neonatal specimens; as = anterior supracallosal sector; ps = posterior supracallosal sector; ^▲^= cortical surface area estimates based on other previously measured brains of similar mass [37]; ^AVG^ = averaged adult data from a previous publication [37]; Family Legend: Bal. = Balaenopteridae; Mon. = Monodontidae; Phoc. = Phocoenidae; Ziph. = Ziphiidae; Kog.= Kogiidae; Delph. = Delphinidae; Hom. = Hominidae. Species legend: *Bp* = *Balaenoptera physalus; Mn = Megaptera novaeangliae; Dl = Delphinapterus leucas; Pp = Phocoena phocoena; Zc = Ziphius cavirostris; Kb = Kogia breviceps; Pc = Pseudorca crassidens; Dd = Delphinus delphis; Tt = Tursiops truncatus; Oo = Orcinus orca; Gm = Globicephala melas; Hs = Homo sapiens.* Source legend: auth=measurements by the authors of this study, others cited by numbers in the reference list.
